# A Health Risk Assessment of Workers Exposed to Organic Paint Solvents Used in the Korean Shipbuilding Industry

**DOI:** 10.3390/toxics12120903

**Published:** 2024-12-11

**Authors:** Sue-Ji Seo, Sae-Mi Shin, Wonsuck Yoon, Sang-Hoon Byeon

**Affiliations:** 1 Health and Safety Convergence Science Introduction, College of Health Science, Korea University, Seoul 02841, Republic of Korea; 2 Research Institute of Health Sciences, Korea University, Seoul 02841, Republic of Korea; 3 Allergy and Immunology Center, Korea University, Seoul 02841, Republic of Korea; 4 Department of Health and Environmental Science, College of Health Science, Korea University, Seoul 02841, Republic of Korea

**Keywords:** risk assessment, paint, solvent, occupational exposure, sensitivity analysis

## Abstract

In the shipbuilding industry, during the painting process, workers are exposed to various substances in paint, including organic solvents that can adversely affect their health. Most workplace exposures to organic solvents involve mixtures of organic compounds. Therefore, in this study, the hazard quotient (HQ) and hazard index (HI) were derived using data from the Workplace Environmental Monitoring Program in Korea for six organic solvents (xylene, n-butanol, ethylbenzene, isobutyl alcohol, toluene, and methylisobutyl ketone [MIBK]) commonly used in the steel shipbuilding industry. The non-carcinogenic risk was assessed using Monte Carlo simulations, and sensitivity analysis was performed using the Spearman rank correlation coefficient with the R program. The HI for neurotoxicity and developmental toxicity exceeded 1 in the 25th and 75th percentile, respectively. According to the sensitivity analysis, the HI for neurotoxicity was correlated with the concentration of xylene and its exposure duration, whereas that for developmental toxicity was correlated with the concentration of ethylbenzene and MIBK and their exposure duration. This study investigated the health risks posed by organic solvents among workers involved in the painting process of shipbuilding. Additional research on percutaneous exposure to organic solvents and a detailed process analysis are needed.

## 1. Introduction

In Korea, the shipbuilding industry has experienced steady growth, driven by the continuous increase in support for international trade [1]. In 2008, it recorded the highest growth rate among all industries, showing a 21.1% increase from the previous year, with a shipbuilding output of 12.47 million compensated gross tons and a workforce exceeding 130,000 [2]. The shipbuilding industry encompasses various processes, including cutting, welding, painting, forging, electroplating, and electrical facility installation and repair. Its manufacturing worksite is considerably larger than that of other manufacturing industries [2].

Painting is an essential process in the shipbuilding industry [3,4]. The ingredients in paint vary depending on various factors, such as the process of handling paint, the purpose of the paint, and the manufacturer of the paint. Consequently, painters are exposed to diverse hazardous substances, including organic solvents [5].

Organic solvents are used extensively in the manufacture of paints, industrial solvents, and adhesives; therefore, the frequency of occupational diseases caused by organic solvents is high among workers working in shipyards involved in several painting processes [5]. Workers exposed to organic solvents may develop neuropsychiatric disorders [6]. For example, acute exposure to high organic solvent levels may induce symptoms related to central nervous system depression, including lethargy, intoxication, headache, and nausea, along with mucosal irritation of the eyes and throat [7]. In severe cases, acute toxic encephalopathy leads to coma, loss of consciousness, convulsions, and even death [7]. Moreover, exposure to organic solvents induces liver toxicity [8]. Some studies have suggested that short-term and repeated prenatal exposure to high solvent concentrations causes growth restriction, malformation, and biological and behavioral developmental disorders in laboratory animals [9]. Additionally, a meta-analysis of epidemiological studies confirmed developmental toxicity in the children of workers exposed to organic solvents, indicated by various adverse effects including liver dysfunction, kidney disease, skin disease, cardiac dysfunction, and occupational cancer [10].

In the shipbuilding industry, painting is performed indoors, outdoors, and on hulls. Because the hull and block are sealed spaces, exposure to high concentrations of organic solvents is possible [11]. In 1996, a worker in Korea was diagnosed with toxic encephalopathy accompanied by moderate cognitive dysfunction after being exposed to organic solvents for approximately 13 years while performing spray and brush painting in a shipyard [7]. One study reported that the neurobehavioral abilities of shipbuilding painters decreased compared to the control group [12], and another study found that long-term exposure to xylene and other mixed organic solvents among shipyard spray painters resulted in impaired motor performance and decreased perception [13]. The prevalence of neuropsychological symptoms in shipbuilding painters was considerably higher than that in the non-painting group [14,15]. Moreover, organic solvents can cross the placenta and negatively affect fetal development, as indicated by a study that found that the children of painters exposed to organic solvents before pregnancy had a higher risk of developing congenital malformations than those of painters who were unexposed [16].

At industrial sites, workers are primarily exposed to mixed organic solvents rather than single substances [17]. The effects of mixed organic solvents on the human body are additive [17]. However, limited research has been conducted on the chemical exposure assessment of mixed organic solvents used in the Korean shipbuilding industry. Additionally, approximately 98.8% of industrial accidents in this sector are related to steel shipbuilding or repair [18]. Therefore, using data from the 2018 Work Environment Monitoring Program (WEMP) within the steel shipbuilding industry, which has a high incidence of occupational diseases in Korea, this study aims to evaluate the health hazards associated with the painting process by determining the hazard index (HI) according to the toxicity of the organic solvents used.

## 2. Materials and Methods

### 2.1. Chemicals and Target Population for Evaluation

In this study, three of Korea’s national statistics were used. The Korean Ministry of Environment provided the chemical substance statistics survey, which we used to select target substances for non-carcinogenic risk assessment [19]. The Korea Occupational Safety and Health Agency’s WEMP results provided the measured values of the target substances [20]. The Korea Occupational Safety and Health Agency’s work environment survey report provided the variables used to calculate the risk assessment [21].

The statistical survey of chemical substances in Korea provides general information on businesses using chemical substances, including data on the maximum storage, stockpiling, and usage of chemical substances, chemical accidents, and the status of handling [19]. There were 13 companies in Korea that were engaged in the steel shipbuilding industry [19]. Among the chemicals used by Korea’s 13 companies, six organic solvents with annual usage and sales exceeding 1000 t and that are subject to WEMP were selected. The six solvents were xylene, n-butanol, ethylbenzene, isobutyl alcohol, toluene, and methylisobutyl ketone (MIBK).

The concentration (daily level concentration) for six organic solvents was obtained from the 2018 WEMP. The WEMP measured the air concentrations of 190 harmful agents in Korean workplaces [20]. The results were then presented to the government to determine if the regulatory limit was exceeded [20]. The WEMP data included the following information: the year of measurement, the business code, the name of the representative, the business address, the industry code, the process code, the name of the worker, the chemical’s name, and the measured value [20,22]. The measurements of six solvents, xylene, n-butanol, ethylbenzene, isobutyl alcohol, toluene, and methylisobutyl ketone (MIBK), were 952, 449, 868, 172, 427, and 290, respectively, totaling 3158 measurement data points.

### 2.2. Health Risk Assessment

In this study, inhalation was assumed to be the primary route of organic solvent absorption. The non-carcinogenic risks from organic solvent inhalation were assessed using the hazard quotient (HQ) and hazard index (HI) [23,24]. The potential non-carcinogenic effect in humans is generally quantified by calculating the ratio of the inhalation exposure concentration (EC) and the Reference concentration (RfC) as in Equation (1) [25]. An HQ exceeding 1 suggests an unacceptable non-carcinogenic risk, whereas an HQ below 1 suggests an acceptable risk [24]. The HI is the sum of the HQ for toxicants that affect the same target organ as in Equation (2) [26]. An HI value exceeding 1 indicates a high probability of adverse health effects for non-carcinogenic hazards. The HI of a mixture of organic solvents can also be compared with that of similar mixtures to prioritize risk management measures [26,27]. Therefore, in this study, the six substances subject to risk assessment were classified according to specific target organ systems, and then, the HQs were summed to calculate the HI for each target system. The HI for neurotoxicity is the sum of the HQ of the neurotoxic solvents: xylene, n-butanol, isobutyl alcohol, and toluene. The HI for developmental toxicity is the sum of the HQ of MIBK and ethylbenzene.

HQ = EC ÷ RfC(1)

HI = ΣHQ(2)

To determine the HQ of the six organic solvents, the inhalation EC was calculated using Equation (3), as follows.

EC = (C × ET × ED × EF) ÷ AT(3)

where EC is the exposure concentration (ppm), C is the daily level contaminant concentration in air (ppm), ET is the exposure time spent in the workplace (hours/week), ED is the exposure duration (years), EF is the exposure frequency for the workplace (days/week), and AT is the averaging time (days). More detailed information is in Table 1.

Among the variables used to derive the EC, daily exposure time, exposure duration, and exposure frequency were derived from the Working Conditions Survey Report [21], the results of anational statistical data survey that comprehensively surveyed the work environment of employed people aged 15 years or older in Korea, including employment type, occupation, industry, and exposure to risk factors [21]. R software (ver. 4.4.0; R Foundation, Indianapolis, IN, USA) was used to select the distribution type with the minimum Akaike Information Criterion and evaluate the parameter’s fit. The representative value and scatter plot were then derived. Consequently, the daily exposure time, exposure duration, and exposure frequency exhibited a logistic, exponential, and log-normal distribution, respectively.

In this study, we used the RfC and Reference Dose (RfD) values (Table 2) suggested by the Environmental Protection Agency (EPA) Integrated risk information system (IRIS) to assess the risk posed by six organic solvents to shipbuilding workers [28,29,30,31,32,33]. In this process, RfD was converted to RfC using Equation (4).

RfC (mg/m^3^) = RfD (mg/kg/day) × 70 kg/20 m^3^/day (4)

Following the EPA IRIS, the RfC of xylene was derived based on a no-observed-adverse-effect level (NOAEL) of 50 ppm, at which impaired motor coordination, a neurological effect, was observed as a critical effect [28]. In addition, the RfD of n-butanol was derived based on a NOAEL of 125 mg/kg/day, at which point, decreased activity and ataxia have critical effects on the central nervous system [29]. The RfD of isobutyl alcohol was derived based on a NOAEL of 316 mg/kg/day, at which, decreased activity and ataxia were shown as critical effects, with a pathway similar to that of n-butanol in the human body [30]. The RfC of toluene was derived based on a NOAEL of 34 ppm, at which, the neurological effects in occupationally exposed workers were critical [31]. The RfC of MIBK was derived based on a NOAEL_HEC_ of 1026 mg/m^3^, at which critical effects, such as decreased fetal body weight, skeletal deformation, and increased fetal mortality, were observed in rats [32]. The RfC of ethylbenzene was derived based on its NOAEL (434 mg/m^3^), which has a critical effect on developmental toxicity [33]. Therefore, the target toxicity was divided into two categories to derive the HI for nervous system development.

### 2.3. Statistical Analysis

The most popular method for minimizing uncertainty in risk assessment is the Monte Carlo simulation, which takes random samples from the population and accounts for the parameter distribution type [34,35]. Since Monte Carlo simulation applies probability distributions to parameters, if we input parameters into the formula 10,000 times, the overall risk we want to know will have a probability distribution, and we can obtain the non-carcinogenic probability for the population. Therefore, we used R software (ver. 4.4.0; R Foundation, Indianapolis, IN, USA) to perform the Monte Carlo simulation in this study. We derived HQ by repeating the Monte Carlo simulation 10,000 times and applying the parameter distribution (Table 1) [22].

We performed a sensitivity analysis using the rank correlation coefficient. Spearman’s rank correlation coefficient measures the sensitivity of parameters [36,37,38,39,40,41,42]. A positive Spearman’s rank correlation coefficient signifies that R(Xi) generally rises as R(Yi) rises [43]. The degree of correlation strength was categorized as follows: 0–0.19, 0.20–0.39, 0.40–0.69, 0.70–0.89, and 0.90–1.0, indicating a negligible, low, moderate, high, and very high correlation, respectively [44].

## 3. Results

### 3.1. Exposure Assessment

The average concentrations and standard deviations of xylene, n-butanol, isobutyl alcohol, toluene, MIBK, and ethylbenzene were 19.66 ± 51.24 ppm, 12.84 ± 29.37 ppm, 4.50 ± 6.62 ppm, 2.50 ± 5.40 ppm, 2.56 ± 4.07 ppm, and 9.44 ± 25.26 ppm, respectively. Detailed box plots for the concentrations are provided in Figure S1.

### 3.2. Non-Carcinogenic Risk

Table 3 presents the non-carcinogenic risk assessment values obtained from Monte Carlo simulations for the six organic solvents. Xylene had an HQ exceeding 1 at the percentile of 29.70, indicating that xylene poses the highest non-carcinogenic risk among the six organic solvents. n-Butanol had an HQ exceeding 1 at the percentile of 60.91. Isobutyl alcohol, MIBK, and ethylbenzene had HQ values exceeding 1 at the percentiles of 82.02, 92.01, and 75.41, respectively. In contrast, toluene had an HQ of 0.34 at the 95th percentile, indicating that it presents the lowest non-carcinogenic risk among the six organic solvents.

As shown in Table 4, HI was calculated as the sum of the HQ for toxic substances with the same target toxicity. The HI for neurotoxicity exceeded 1 at the percentile of 15.17, indicating an unacceptable risk. The HI for developmental toxicity exceeded 1 at the percentile of 67.95, indicating an unacceptable risk.

### 3.3. Sensitivity Analyses

Table 5 shows the contribution of the exposure factors to the non-carcinogenic risk of the six organic solvents. When estimating the non-carcinogenic risk, the chemical concentration showed the strongest correlation at 0.85–0.91, followed by the exposure duration (years of work) with a moderate correlation at 0.36–0.47. The daily exposure time and exposure frequency showed negligible correlations of less than 0.19. Table 6 shows the contribution of exposure factors to HI in the nervous system. Among the four organic solvents used to derive the HI for the nervous system, the concentration of xylene showed a moderate correlation at 0.65, and n-butanol, isobutyl alcohol, and toluene, excluding xylene, showed weak correlations. The exposure duration showed a moderate correlation (0.56), and the daily exposure time and exposure frequency showed weak correlations. Table 7 also shows the contribution of exposure factors to the HI for developmental toxicity. Ethylbenzene, MIBK, and exposure duration showed moderate correlations of 0.56, 0.44, and 0.55, respectively, whereas daily exposure time and exposure frequency showed weak correlations.

## 4. Discussion

This study assessed workers’ exposure to specific organic solvents used in the shipbuilding painting process in Korea in 2018 and evaluated the resulting health risks. Using Korea’s national statistical data, we identified the six most commonly used organic solvents in the shipbuilding industry and conducted health risk assessments for each chemical substance. In addition, considering the characteristics of mixed, combined, and exposed organic solvents, we conducted a toxicity risk assessment based on the EPA IRIS toxicity classification to determine the level of risk caused by organic solvents for workers in the shipbuilding painting process in Korea.

The results of the risk assessment indicate that when workers were exposed to mixed organic solvents, the probability of the HI for each target toxicity exceeding 1 was the 16th percentile for neurotoxicity and the 68th percentile for developmental toxicity. Chronic exposure to mixed organic solvents among shipyard painters has raised concerns regarding central nervous system injury, which is characterized by abnormal speed and coordination of movement [45]. In a study involving 41 workers from four small factories using mixed organic solvents, symptoms of postural sway with eyes open, due to the adverse effects of organic solvents on the nervous system, were significantly higher in the experimental group than in the control group [46]. Additionally, workers exposed to mixed organic solvents may experience adverse effects on the nervous system even at low exposure levels [46]. This suggests that the HI for the nervous system, which can cause adverse effects even at low exposure levels, presents a relatively higher risk than the HI for developmental toxicity.

Although the proportion of workers with an HI greater than 1 was lower than for neurotoxicity, our study found that developmental toxicity was a risk for the 32nd percentile of workers. Occupational exposure to organic solvents increased the risk of major malformations in women 13 times, indicating a dose–response relationship [47]. In addition, a study measuring the risk of the reproductive or developmental effects on paint workers working 200 days a year for 40 years estimated that seven inhaled organic chemicals had an HQ of 1 or higher, with five organic chemicals having an HQ of 10 or higher and one organic chemical having an HQ of 100 or higher [48]. The findings of this study establish that workers involved in the shipbuilding painting process are susceptible to neurotoxicity and developmental toxicity from mixed organic solvents, necessitating ongoing monitoring of exposure and risk levels and the implementation of targeted risk reduction measures.

The highest average concentration was xylene (19.66 ± 51.24 ppm), followed by n-butanol (12.84 ± 29.37 ppm), ethylbenzene (9.44 ± 25.26 ppm), isobutyl alcohol (4.50 ± 6.62 ppm), MIBK (2.56 ± 4.07 ppm), and toluene (2.50 ± 5.40 ppm). Xylene is the solvent that workers are most frequently exposed to during the painting process [11]. As suggested by Koh et al. [11], the arithmetic mean of xylene was 21.74 ppm, and the geometric mean was 12.81 ppm for spray painting, while the arithmetic mean was 18.54 ppm, and the geometric mean was 11.82 ppm for brush painting [11]. In addition, according to a study that measured the air concentration of organic solvents at the workplace of 180 shipbuilding painters in Korea, the average concentration of xylene, MIBK, ethylbenzene, n-butanol, and toluene was 22.70 ppm, 10.04 ppm, 8.26 ppm, 7.34 ppm, and 3.70 ppm, respectively [45]. In view of this, the concentration of xylene is consistently high in shipbuilding painting processes, which is consistent with our results. These results are because xylene is an organic solvent that is widely used regardless of the type of paint and exists in a content range of 1 to 45% [49]. Also, because shipyards must use organic solvents in very confined spaces, the risk of inhaling solvents is higher than that in open spaces [50]. Furthermore, the actual exposure pattern of workers to organic solvents did not remain constant over time, with high concentrations observed during painting and low concentrations observed while moving around the workplace or preparing for painting [51].

The results of the sensitivity analysis provide a useful reference for personal exposure management in terms of activity patterns because parameters such as daily exposure time and exposure duration can affect worker inhalation [52]. When estimating the non-carcinogenic risk, chemical concentration showed the strongest correlation, followed by exposure duration. The airborne concentrations of organic solvents had the greatest influence on non-carcinogenic risk. HQ and HI showed the strongest correlation with airborne organic solvent concentration. Specifically, only the concentration of xylene, which constitutes the largest proportion of paint materials, was correlated with the HI for the nervous system. Three main types of paint materials are used in the painting process of the shipbuilding industry: paint, thinner, and hardener [49,53]. The main organic solvent component in these materials is xylene [49,53]. In addition, toluene, isopropyl alcohol, and trimethylbenzene are present in certain products [53]. Xylene was detected most frequently in a study that measured mixed organic solvents in the air inhaled by 54 workers in the painting departments of three shipyards, followed by n-butanol, toluene, ethylben-zene, and MIBK [53].

Usually, the levels of impact, signs, and symptoms caused by chemicals vary depending on their concentration [54]. However, even if the concentration of solvents in the workplace is not high, continuous exposure can cause health problems owing to night work and the use of other toxic chemicals [50]. In addition, shipyards often operate in confined spaces where solvent concentrations may not always be measurable; thus, personal protective measures must be actively used [50].

Nonetheless, the WEMP data used in this analysis are limited in their ability to comprehensively represent the working conditions of specific workplaces in Korea. Because the measurements are only taken twice a year, this may reduce their representativeness. Therefore, further research is needed to complement the data on the working environments of individual workplaces. The work environment data used in this study are a composite of records from various measuring technicians and have the limitation that this study evaluates various painting tasks, such as brush painting and spray painting, as one group without dividing them into detailed tasks [11]. In addition, the WEMP data used in this study measured airborne pollutants and did not consider dermal exposure. However, during the painting process, workers may experience significant skin exposure to paint aerosols, fog, or droplets settling on their clothes or skin [55]. Therefore, additional research on percutaneous exposure to organic solvents in the shipbuilding industry is required.

## 5. Conclusions

This study was aimed at evaluating the non-carcinogenic risk of six organic solvents widely used in the Korean shipbuilding painting process, deriving an integrated risk index based on toxicity, and assessing the risk associated with mixed organic solvents.

According to the non-carcinogenic health risk assessment, xylene had an HQ exceeding 1 at the percentile of 29.70, and n-butanol had an HQ exceeding 1 at the percentile of 60.91. Isobutyl alcohol, MIBK, and ethylbenzene exhibited HQ exceeding 1 at the percentiles of 82.02, 92.01, and 75.41, respectively. On the other hand, toluene had an HQ of 0.34 at the 95th percentile, showing the lowest non-carcinogenic risk among the six organic solvents. The results of the sensitivity analysis showed that the concentration of organic solvents had the most significant effect on non-carcinogenic risk. Thus, the concentrations of the organic solvents and risk ranking are identical.

The HI for neurotoxicity exceeded 1 at the percentile of 15.17, indicating an unacceptable risk, whereas the HI for developmental toxicity exceeded 1 at the percentile of 67.95, indicating an unacceptable risk. These results are similar to those of previous studies showing that mixed organic solvents exhibit neurotoxicity and developmental toxicity.

Future studies should focus on the detailed classification of various shipbuilding painting processes by workspace, painting sequence, painting materials, and exposure to organic solvents due to percutaneous exposure during the painting process.

## Figures and Tables

**Table 1:** Variables of exposure used for risk assessment.

Parameter	Definition	Value	Distribution Type	Unit
C	Concentration (daily level concentration)	As shown in Figure S1.	Log-normal	ppm
ET	Daily exposure time	8 (±1.11)	Logistic	hours/week
ED	Exposure duration	11 (±8.69)	Exponential	years
EF	Exposure frequency	268 (20.92)	Log-normal	days/week
AT	Average lifetime	69,408	Fixed value	days

**Table 2:** RfC and RfD of six organic solvents used in shipbuilding.

	Toxicity Effect	
	RfC (mg/m^3^)	RfD (mg/kg/day)
Xylene	Nervous	Other (Decreased body weight, increased mortality)
	1.00 × 10^−1^	2.00 × 10^−1^
N-Butanol	-	Nervous
	-	1.00 × 10^−1^
Isobutyl alcohol	-	Nervous
	-	3.00 × 10^−1^
Toluene	Nervous	Urinary
	5.00	8.00 × 10^−2^
MIBK	Developmental	-
	3.00	-
Ethylbenzene	Developmental	Hepatic, Urinary
	1.00	1.00 × 10^−1^

**Table 3:** Hazard quotient (HQ) of six organic solvents.

Substance	HQ				HQ > 1 (Percent)
	25th Percentile	50th Percentile	75th Percentile	95th Percentile	
Xylene	7.27 × 10^−1^	3.65	19.26	176.29	70.30
n-Butanol	7.69 × 10^−1^	4.89 × 10^−1^	3.04	37.88	39.09
Isobutyl alcohol	7.17 × 10^−3^	5.97 × 10^−2^	4.79 × 10^−1^	8.96	17.98
Toluene	1.41 × 10^−3^	7.36 × 10^−3^	3.67 × 10^−2^	3.41 × 10^−1^	1.83
MIBK	2.13 × 10^−3^	1.63 × 10^−2^	1.13 × 10^−1^	1.93	7.99
Ethylbenzene	3.38 × 10^−2^	1.83 × 10^−1^	9.69 × 10^−1^	9.92	24.59

**Table 4:** Hazard index (HI) by target toxicity.

Toxicity Effect	HI				HI > 1 (Percent)
	25th Percentile	50th Percentile	75th Percentile	95th Percentile	
Nervous	2.11	8.82	35.37	257.41	84.83
Developmental	7.32 × 10^−2^	3.36 × 10^−1^	1.58	13.19	32.05

**Table 5:** Sensitivity analysis of factors that potentially contribute to HQ.

Risk Factors	Spearman’s Rank Correlation Coefficient					
	Xylene	n-Butanol	Isobutyl Alcohol	Toluene	MIBK	Ethylbenzene
Concentration	8.55 × 10^−1^	8.79 × 10^−1^	9.08 × 10^−1^	8.47 × 10^−1^	8.98 × 10^−1^	8.60 × 10^−1^
Exposure duration	4.70 × 10^−1^	4.30 × 10^−1^	3.65 × 10^−1^	4.72 × 10^−1^	3.98 × 10^−1^	4.56 × 10^−1^
Daily exposure time	4.72 × 10^−2^	2.82 × 10^−2^	4.70 × 10^−2^	3.79 × 10^−2^	2.66 × 10^−2^	4.91 × 10^−2^
Exposure frequency	2.72 × 10^−2^	2.09 × 10^−2^	4.02 × 10^−2^	4.07 × 10^−2^	2.95 × 10^−2^	4.49 × 10^−2^

**Table 6:** Sensitivity analysis of probable factors attributed to neurotoxicity based on HI.

Risk Factors		Spearman’s Rank Correlation Coefficient
		HI_Nervous System Toxicity
Concentration	Xylene	6.52 × 10^−1^
	n-Butanol	2.51 × 10^−1^
	Isobutyl alcohol	1.00 × 10^−1^
	Toluene	3.43 × 10^−2^
Exposure duration		5.60 × 10^−1^
Daily exposure time		5.12 × 10^−2^
Exposure frequency		3.31 × 10^−2^

**Table 7:** Sensitivity analysis of probable factors attributed to developmental toxicity based on HI.

Risk Factors		Spearman’s Rank Correlation Coefficient
		HI_Developmental Toxicity
Concentration	MIBK	4.41 × 10^−1^
	Ethylbenzene	5.66 × 10^−1^
Exposure duration		5.53 × 10^−1^
Daily exposure time		4.93 × 10^−2^
Exposure frequency		4.57 × 10^−2^

## Data Availability

The datasets presented in this article are not readily available because of information security issues.

## Supplementary Materials

The following supporting information can be downloaded at: https://www.mdpi.com/article/10.3390/toxics12120903/s1, Figure S1: Concentration characteristics of six organic solvents.
