# Supplementary material for: A Health Risk Assessment of Workers Exposed to Organic Paint Solvents Used in the Korean Shipbuilding Industry
Source: Toxics. 2024 Dec 11;12(12):903. doi: 10.3390/toxics12120903 (PMC11728811; doi:10.3390/toxics12120903)
Supplement: Supplementary file 1 [file toxics-12-00903-s001.zip › toxics-3284418-supplementary.pdf]

## Supplementary material to:

# Health risk assessment of workers exposed to organic paint solvents used in the Korean shipbuilding industry

Sue Ji Seo [1], Sae Mi Shin [2], Won Suck Yoon [3*], Sang Hoon Byeon [1,4,*]

[1] Health and Safety Convergence Science Introduction, College of Health Science, Korea University, Seoul 02841, Republic of Korea

[2] Research Institute of Health Sciences, Korea University, Seoul 02841, Republic of Korea

[3] Allergy and Immunology Center, Korea University, Seoul 02841, Republic of Korea

[4] Department of Health and Environmental Science, College of Health Science, Korea University, Seoul 02841, Republic of Korea

* Correspondence: shbyeon@korea.ac.kr

## Contents

1. The concentraion of six organic solvents ........................................................................................ 2

Figure S1 shows the daily level concentration of six organic solvents used in the shipbuilding painting process with the y-axis on a logarithmic scale.

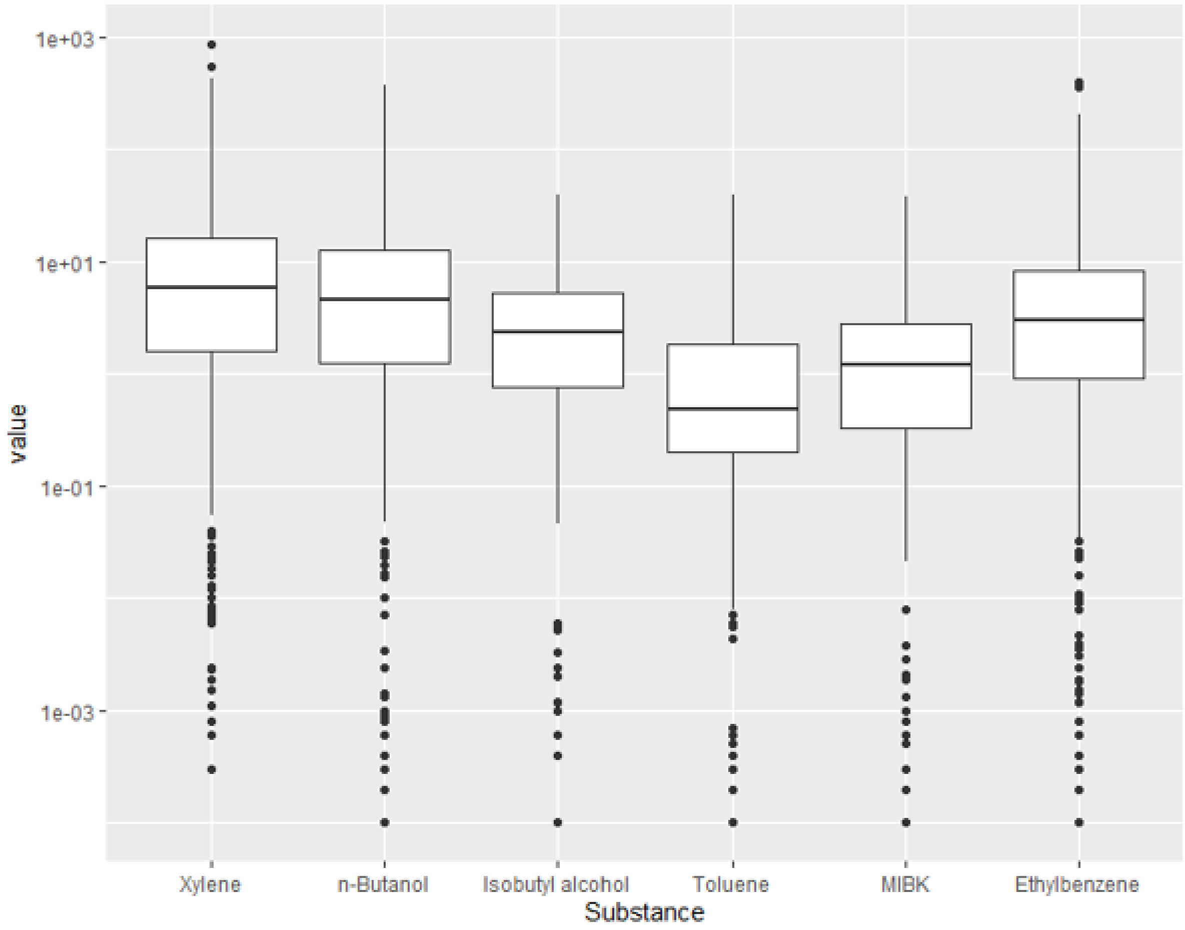


**Figure S1.** Concentration characteristics of six organic solvents.
